# Is Gastric Involvement by *Strongyloides stercoralis* in an Immunocompetent Patient a Common Finding? A Case Report and Review of the Literature

**DOI:** 10.1007/s11686-021-00438-9

**Published:** 2021-06-18

**Authors:** Irene Pecorella, Tom Richard Okello, Gaia Ciardi, David Martin Ogwang

**Affiliations:** 1 Department of Radiological, Oncological and Anatomic Pathology Sciences, University of Rome “La Sapienza”, Viale Regina Elena, 324, 00161 Rome, Italy; 2 St Mary’s Hospital Lacor, Gulu, Uganda; 3 Department of Surgery, Gulu University Medical School, Gulu, Uganda

**Keywords:** *Strongyloidesstercoralis*, Roundworm, Intestinal nematode

## Abstract

**Purpose:**

Gastric infection with *Strongyloides stercoralis *(*SS*) usually occurs in immunocompromised patients. The unexpected observation of this parasite in an otherwise healthy young lady who had undergone upper endoscopy and biopsy sampling of the gastro-duodenal mucosa, prompted us to review the literature to ascertain the conditions favouring gastric colonization by *SS.*

**Methods:**

Pathology files of gastroduodenal biopsies received at St. Mary’s hospital, Northern Uganda, between 2007 and 2017 were reviewed. Pubmed search was performed under the headings “*Strongyloides stercoralis*”, “Gastric parasitosis”.

**Results:**

Histology of the only gastroduodenal biopsy with SS infection showed parasite eggs, immature rhabditiform larvae, and numerous adult worms in gastric pits and rhabditiform larvae in interepithelial parasitic tunnels, causing reactive changes of the glandular epithelium. There was no significant acute inflammatory cell infiltrate surrounding the parasites. Literature review showed that gastric *SS* infection appears to be very uncommon and was, as expected, largely prevalent in immunodeficient individuals (84.2% of published cases). The rare gastric *SS* infection is a complication of systemic strongyloidiasis, either hyperinfective, or disseminated form. It is also commonly associated with duodenal infection at microscopical examination.

**Conclusion:**

Involvement of gastric mucosa in the absence of duodenal strongyloidiasis appears to be quite rare and false-negative histopathological exams are reported if only the stomach is biopsied.

## Introduction

Strongyloidiasis is an endemic disease in Africa, South America, and Southeast Asia, caused by soil-transmitted nematodes in the genus *Strongyloides,* particularly *S. stercoralis* (*SS*)*.* The other species, *S. fuelleborni*, is found sporadically in Central Africa and Papua New Guinea and may produce limited infections in humans [1]. As for other helminthic infections, strongyloidiasis is associated with low socioeconomic conditions, poor hygiene, and ineffective health care facilities. Strongyloidiasis is an emerging disease in even nonendemic regions due to *SS-*infected people emigrating from endemic to developed countries.

Free-living *SS* larvae exist in two forms: rhabditiform and filariform infective larvae. Free-living rhabditiform larvae are passed in the stool and can become either infective filariform larvae (direct development) or adult male and female worms that mate and produce eggs in contaminated moist soil. Open defecation and walking barefoot are at risk for infection, as *SS* filariform larvae penetrate the skin of the human host to reach into the venous microcirculation via lymphatics. From the blood, the larvae transmigrate into the alveoli of lungs, trachea, where they are eventually coughed up and swallowed. From the pharynx, oesophagus, and stomach, the parasites finally reach into the duodenum and upper jejunum where they burrow into the mucosa and mature to become adult female worms [1]. There, they produce by parthenogenesis up to 40 eggs/day that hatch into rhabditiform larvae, which are released into the lumen of the intestine and are excreted in stool for free-living cycle. Invasive third-stage, filariform larvae can be also ingested in contaminated water. Lung migration is unnecessary if juveniles in food or water are directly swallowed and conveyed to the small intestine [2]. Human to human spread has been reported after anal or oral sexual contact [2, 3]. Although most of the larvae will be excreted in the stool, delayed defecation or constipation can induce in some cases the parasites to molt to infective filariform larvae in the intestine and immediately re-infect the host by penetrating the bloodstream either in the intestinal wall, or the perianal skin (autoinfection). Larva currens (racing larvae) is the pathognomonic cutaneous manifestation of *SS* infection that usually occurs during an external autoinfection episode. The serpiginous urticarial rash is caused by rapid (approximately 15 cm/h) moving of *SS* larvae from the anal area down the upper thighs [4].

In contrast to other nematodes—which transform into infective filariform larvae outside the host—*SS* is the only helminth capable of completing the cycle within the host. Without treatment*,* autoinfection is responsible for the perpetuation of the parasite even after a long period after original infection, as subjects may mount a partially effective immune response, unable to eradicate the infection, but containing the intensity of infection (chronic strongyloidiasis). On the other hand, heavy invasive filariform larvae dissemination to the sites in which *SS* is located during its life cycle, i.e., the proximal small bowel, colon, and lungs (hyperinfection syndrome), or to any organ (disseminated *SS*) occurs in immunosuppressed individuals, due to enhancement of autoinfection cycle, resulting in potentially life-threatening complications. Therefore, adequate diagnosis and treatment are critical in patients at high risk for complications due to *SS* infection.

In immunocompetent individuals, *SS* can rarely inhabit the stomach, mostly when reduction of gastric acid secretion is present.

Herein, we present a case of *SS* gastric infection in an immunocompetent Ugandan female patient, diagnosed with mucosal biopsy of stomach and duodenum. Review of the literature on *SS* gastric infection will also be presented.

## Material and Methods

**Fig. 1:**
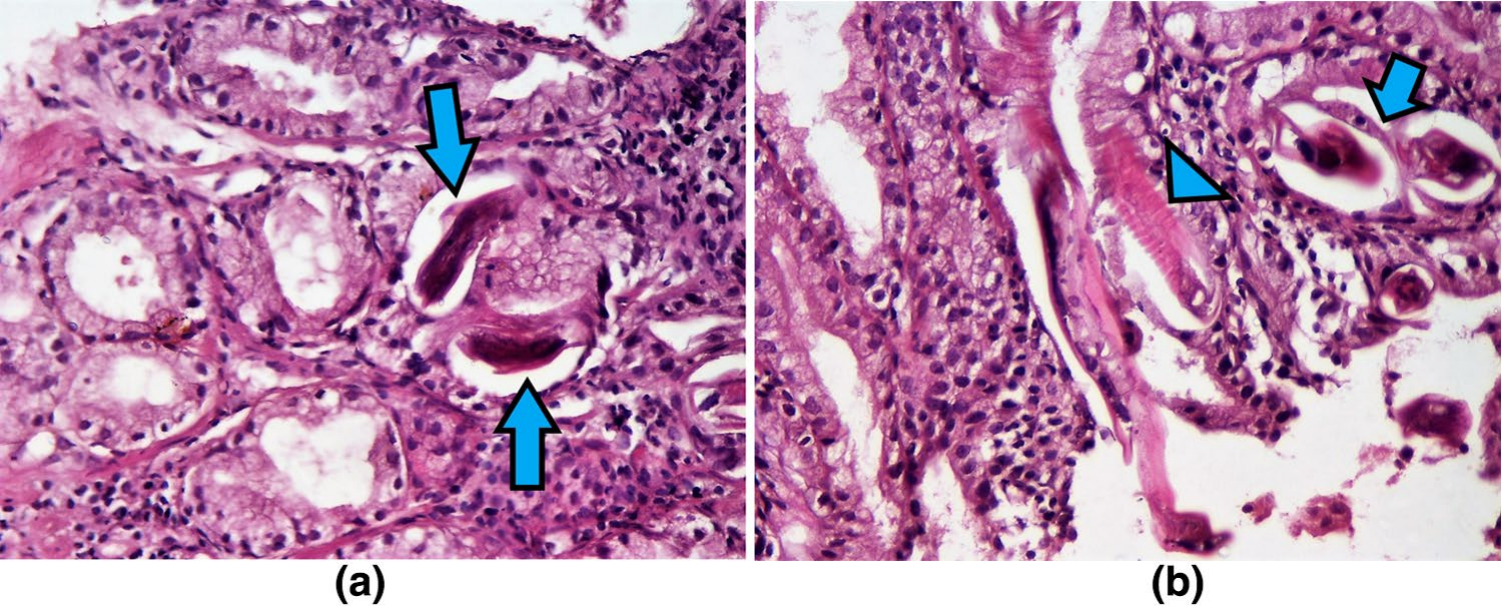
**a**, **b** *Strongyloides stercoralis* rhabditiform larvae (arrows) in the gastric antral glands. The larvae are small, although some can reach about 1.5 mm in length. The parasitic female worm measures 2–3 mm and shows fine transverse striations (arrowhead). (Haematoxylin–eosin, × 250)

**Fig. 2:**
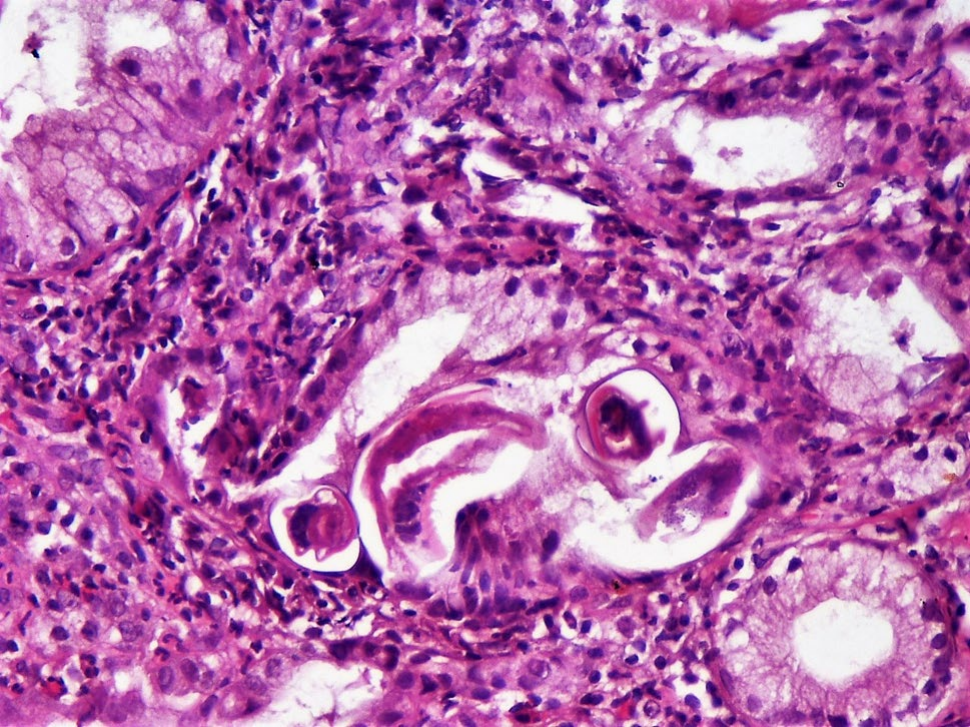
Disruption of the epithelial lining of the gastric glands results from larval's entrance and exit from the parasitic tunnels. (Haematoxylin–eosin, × 250)

A 30-year-old female, living in a rural area in North Uganda, presented at Lacor St. Mary’s Hospital of Gulu with a history of dysphagia in the last 3 months. Clinical history revealed also complaints of chronic episodic diarrhea and abdominal pain. The physical examination was normal except for epigastric tenderness. At endoscopy, haemorrhagic areas were noted in the antral and duodenal mucosa. Biopsies of these areas were taken and submitted for pathological examination. Microscopically, the gastric antral mucosa revealed superficial ulcerations and nonspecific chronic inflammation with scattered eosinophils in the lamina propria. Parasite eggs, immature rhabditiform larvae, and numerous adult worms showing bulbous enlargement at oesophageal end and slender tail end, conforming to *SS,* were present in gastric pits (Fig. 1a,b). Parasites were also observed in interepithelial parasitic tunnels (Fig. 2), where developing adult parasitic females burrow to deposit their ova. There was no significant acute inflammatory cell infiltrate surrounding the parasites. Severe reactive changes of the gastric epithelium were noticed. Parasites were also present in the duodenal mucosa, where reactive changes of the glandular epithelium were more apparent. The patient received ivermectin 200 μg/kg for 2 days and subsequent stool examination was negative for ova and parasites. Blood tests to evaluate eosinophilia were not performed.

Results of a comprehensive search of the literature database, conducted to analyse the incidence, and the endoscopical features of *SS* gastric infection, are summarised in Table 1.

**Table 1:** Gastric *Strongyloides stercoralis* infection in the literature

Reference	Age	Gender	Extra-gastric	Gastric site	Co-morbidities	Follow-up
[3]	72	♂	Ni (normal endoscopy)	Antrum and corpus	Bronchiolar asthma, heavy drinker	Cured with mebendazole 200 mg/d orally for 2 weeks
[6]	67	♀	ni	Nr	Diabetes and immune thrombocytopenic purpura on corticosteroid treatment since 3 months	Started with oral albendazole. Died of sepsis
[20]	57	♂	Small bowel	Lesser curvature	Rejection of corneal transplant treated with corticosteroids	Cured with thiabendazole, 1.5 g/kg twice daily for 3 days
	57	♀	Lung, liver, small intestine		Cellular immunity deficiency, recent cryptococcal meningitis treated with antibiotics and corticosteroids	Undiagnosed. DOD
[16]	68	♀	Duodenum	Antrum	Rheumatoid arthritis	Improved with mebendazole, lost at follow-up
[17]	43	♀	Duodenum (endoscopically normal)	Fundus and corpus	Mild and intermittent asthma (SS-related), HP infection	Cured with ivermectin 200 {mg/kg orally for 2 weeks
[21]	52	♂	Duodenum	Diffuse	Hairy cell leukemia, type 2 diabetes mellitus	Cured with albendazole 400 mg orally twice daily for 10 days
[22]	68	♀	Duodenal bulb	nr	RA and bronchial asthma on corticosteroid + methotrexate	Cured with ivermectin
[23]	74	♂	Duodenum	Antrum and corpus	Chronic obstructive pulmonary disease Recent episode of mycoplasmal pneumonia	Cured with thiabendazole 1.5 g b.i.d, for 2 days
[24]	70	♂	nr	Large antral ulcer	Ischaemic cardiomyopathy, heavy smoking, large oral cancer 3 mo earlier	Cured with 50 mg/kg thiabendazole for 3 days. Died of metastatic cancer
[25]	52	♂	nr	Antrum	None	Improvement with albendazole 400 mg/d for 3 days
[26]	78	♂	ni	Antrum and corpus	Diabetes	Treatment refused. No follow-up
[27]	74	♂	Duodenum (thickened wall at US)	Antro-pyloric	COPD treated with corticosteroid; pyogenic meningitis	Improved with ivermectin, lost at follow-up
[28]	51	♂	Duodenum	Antrum and fundus	Heart transplant on immunosuppressants	DOD
[29]	74	♂	Heart, small and large intestine	nr	Bronchial asthma on alternate-day 20 to 40 mg prednisone	Thiabendazole, 1.5 g twice daily. DOD
[30]	33	♂	Proximal duodenum	Antrum	Pemphigus vulgaris on corticosteroids	nr
[31]	64	♀	nr	nr	Arthritis	DOD
[32]	53	♂	Colon	Antrum and corpus	Alcohol abuse, HTLV-1 infection	Cured with a single dose of ivermectin (12 mg), followed by thiabendazole 1,5 g/d for 30 days
[33]	79	♀	Entire colon except the rectum Duodenum: ni	Antrum	Monoclonal gammopathy of undetermined significance	Cured, treatment not specified
[34]	33	♀	Duodenum	nr	HIV +	DOD
[35]	30	♂	Duodenum	Antrum and corpus	HIV +	Cured with thiabendazole
[36]	57	♂	Duodenum	Antrum and corpus	CHT for gastroduodenal mantle cell lymphoma 9 mo previously	Ivermectin (200 μg/ kg/d for 10 days. DOD
[37]	45	♀	None	Antrum	Ulcerative colitis on corticosteroids	Cured with albendazole (800 mg/ day/10 days
[38]	35	♂	Ni		Paroxysmal nocturnal haemoglobinuria	Albendazole 400 mg twice/day/ 7 days. Died of septicaemia
	43	♂	Ni	A	HIV + , diffuse large B-cell lymphoma treated with CHT	Improved with albendazole
[39]	68	♂	Duodenum	Diffuse	None	Cured with ivermectine
[40]	34	♀	Duodenum	Nr	SLE treated with corticosteroids and cyclophosphamide	Cured with ivermicine 200 µg/day/9 days
[41]	37	♀	Nr	Corpus	None	No FU
[42]	5	♂	Ileum, colon, lung	Diffuse	Marasmic kwashiorkor	Died
[43]	81	♂	Duodenum	Nr	Arterial hypertension, diabetes mellitus, early gastric cancer	Cured with albendazole (400 mg twice/day/7 days
[44]	83	♂	Duodenal bulb	Antrum	Arterial hypertension and asthma. No corticosteroids	Cured with albendazole
[45]	68	♂	Systemic	Nr	Congestive heart failure, CMV + , HTL-1 +	DOD
[46]	39	♀	Duodenum	Diffuse suppurative ulcers	On corticosteroids for Bell’s palsy since 2 months	
[47]	43	♂	Duodenum, oesophagus	Antrum	Behcet’s syndrome on corticosteroids	Cured with thiabendazole (25 mg/kg/twice/day)
[48]	32	♂	Ni	Nr	AIDS	Cured with albendazole 400 mg/d/3 days
[49]	61	♂	Duodenum	Antrum and corpus	Asthma under corticosteroids	Improvement with thiabendazole
[50]	76	♂	Ni	Pyloric canal	Polymyosistis on corticosteroids	Cured with a combination of parenteral ivermectin and oral albendazole for 2 weeks
[51]	63	♂	Duodenum	Corpus	Mental illness	Cured with thiabendazole 1.5 g/twice/d/4 days

*COPD* chronic obstructive pulmonary disease, *RA* rheumatoid arthritis *US* ultrasound scan, *DOD* died of disease, *ni* not investigated, *nr* not reported, *HP* Helicobacter pylori, *SLE* systemic lupus erythematosus, *CHT *chemotherapy

## Discussion

Strongyloidiasis infects up to 21% of the population in tropical and subtropical areas of Africa, and is commonly found in Asia, and south-America (Argentina, Perù, Brazil), as well as in the south-eastern United States, where 2.5% of the population are infected [2, 4]. A meta-analysis of many reports of strongyloidiasis worldwide showed a prevalence in India of 6.6% in community-based surveys and 11.2% in hospital-based surveys [5]. Community-based surveys, based on foecal tests, clinical presentations, and duodenal lavage tests, showed a prevalence rate of more than 90% in countries like Dominica, Namibia, Papua New Guinea, Gabon, and Israel [6], while a prevalence of less than 5% was reported in Burundi, Central African Republic, Nicaragua, Oman, Republic of Korea, Turkey, America, Venezuela, Vietnam, Sudan, Honduras, Haiti, Grenada, Honduras, Iran, Jordan, Mexico, and Martinique [6]. Reports on *SS* infection rates are, however, hardly comparable and mostly apply diagnostic methods that are inappropriate for detecting the parasite, such as coprologically analysed stool samples [5]. A recent study based on literature data estimated a 8.1% global prevalence and a 10.3% prevalence for the African sub-saharian region using a spatiotemporal statistical modelling approach [7].

Infection is usually associated with agricultural activity. This condition affects 50 to 100 million people worldwide [8], and approximately 60% of infected individuals present with the chronic and asymptomatic form of the parasitic disease [9]. There are no published data concerning the prevalence of such infection in Uganda. We only had one case diagnosed microscopically out of 20% biopsy rate patients undergoing GI endoscopy over a period of 10 years. It should be noted that over 1200 upper gastro-intestinal endoscopies are performed yearly at the St. Mary’s Hospital Lacor [10]. The female gender, youth and adults who are poor peasants predominate amongst patients with upper digestive tract symptoms requiring esofago-gastric-duodenal endoscopy (EGDS) at Lacor Hospital [10]. However, as over 50% of patients infected with the parasite report no symptoms [4], or do not seek medical care due to poverty or distance from hospitals, it is possible that we are underestimating the real prevalence of this disease in our area.

Benevides dos Santos and coll found that < 1% of 1010 duodenal biopsies performed in 14 years contained this parasite in Northern Brazil [11]. High-prevalence areas do seem to exist, however, although cross-reactivity with other helminthic antigens using serological diagnostic methods may account for over-reporting *SS* infection [5].

According to Obiajuru and Adogu [12] who screened 1615 stool samples in south eastern Nigeria for the presence of parasites, upper gastrointestinal ulcerative diseases may be associated with a higher rate of *SS* infection, as the parasite was detected in 2.6% of their duodenogastric ulcer patients, and 0.7% of the non-ulcer subjects. The association with mucosal ulcers can be explained by either the cytotoxic side-effects on the gastrointestinal epithelium of the granules released by the infiltrating eosinophils, which may result in multiple ulcer formation, or by the mechanical trauma caused by rhabditiform larvae burrowing into and exiting from the gastrointestinal mucosa. Occasionally, mucosal ulceration may result in bowel perforation.

Symptomatic individuals commonly develop unspecific gastrointestinal complaints, such as abdominal pain, intermittent episodes of diarrhoea and constipation, nausea, vomiting and, in cases of extensive infection, intestinal obstruction, gastrointestinal bleeding, malabsorption, steatorrhoea, severe pneumonia, septicaemia and weight loss, about 2 weeks after infection. Heavy small bowel worm burdens may themselves produce a protein-losing enteropathy, thus further impairing the host’s immune response to the already present infection. Damage to the intestinal mucosa can cause transmigration of the bacteria and lead to gram-negative sepsis. Dry cough, haemoptysis, rashes arthritis, kidney problems, and heart conditions are also possible in disseminated infection. Pulmonary symptoms are rare in uncomplicated strongyloidiasis, but cough, wheezing and dyspnoea may be part of initial presentation (Löffler’s syndrome) [4]. As *SS* is difficult to diagnose clinically, laboratory methods are commonly used to confirm diagnosis, by the detection of filariform larvae in faecal samples or other body fluid samples of these patients. Positive results are obtained after 3–4 weeks of infection. There is inherent difficulty in identifying *SS* in stool specimens by microscopical examination alone, related to the morphologic similarities that exist between the ova and rhabditiform larvae of *SS* and the ova and rhabditiform larvae of the hookworms *Necatur americanus* and *Ancylostoma duodenale*, which also parasitize the small intestine [2]. Furthermore, the larval load is very low, as larval output is irregular and diagnostic accuracy by stool examination is no higher than 46%, even using DNA-based, highly specific methods, such as real-time PCR [1]. Enzyme-linked immunosorbent serological assay (ELISA), using crude SS somatic antigen, has been reported to have sensitivities of 80–95% [13], though cross-reactions with other nematode parasite infections in *SS* endemic areas cause a number of false positive results. Agar plate culture is considered more efficient than other conventional methods in the parasitological diagnosis of *S. stercoralis* with high sensitivity. Other than real-time PCR, which is often not available and limited by the cost in rural African areas, duodenal aspiration or endoscopic biopsy can be used, the latter showing a histopathologic yield for identifying larvae of 71.4% [14]. The examination of a duodenal aspirate for ova and larvae is also a sensitive diagnostic procedure, unless the infection is in the early stage, with a false-negative frequency of less than 10% [4]. In cases of disseminated infection, the parasite can be identified in sputum, broncho-alveolar lavage, cerebrospinal fluid, skin, urine, and ascites, as well. Eosinophilia is only common in immunocompetent persons.

As Uganda is a country of endemic HIV infection, it is important to underline that patients with advanced HIV and *SS* coinfection may, as well, fail to respond to the standard course of thiabendazole or ivermectin treatment and may die due to disseminated infection [15]. Therefore, secondary prophylaxis with a dose of 200 µg/kg/orally of ivermectin every 2 weeks is recommended until the immune reconstitution associated with HAART occurs.

Bangs and coll consider that gastric mucosal invasion by *SS* is not that unusual, but seldom reported [16]. However, the worm usually resides in the mucosa of the duodenum and upper jejunum, and the stomach is apparently not a congenial site for *SS,* unless infection is favoured by reduced gastric secretion, sometimes caused by iatrogenic chronic acid suppression [17] or intestinal metaplasia of the ossinthic glands. In such instances, the organisms may reach the stomach of the patient via swallowing or via retrograde migration from the proximal small intestine. Our experience seems to indicate that gastric infection is utterly rare in Northern Uganda. Reactive changes of the glandular epithelium were noted microscopically in the present case, but there was no evidence of intestinal metaplasia indicating chronic atrophic gastritis.

Involvement of gastric mucosa in the absence of duodenal strongyloidiasis also appears to be quite rare and false-negative histopathological exams are reported if only the stomach is biopsied [11]. De Paoli and coll showed that even in gibbons dying from SS hyperinfection, the load of parasites in the stomach, when present, was never significant [18]. Random sampling of the gastric mucosa could sometimes account for negative results, due to the sparse distribution of the parasites. Nevertheless, diagnosis of infection by *SS* was made in our patient after demonstrating the parasites in her gastroduodenal biopsies. Apparently, she was neither immunocompromised, nor on prolonged acid suppression treatment. Consequently she lacked the main causes of gastric *SS*, i.e., hyperinfection and gastric achlorhydria. Our literature search retrieved only 38 reports of *SS* infection in the stomach (Table 1). The great majority of these patients (84.2%) showed co-morbidities affecting the immune system, such as cancers or autoimmune diseases, or had recently been under corticosteroids. Overall, only six instances (excluding the present case) of gastric *SS* infection in uncompromised patients have been so far reported, accounting for 15,8% of the published 38 gastric *SS* cases. These included two females, aged 37 and 43 years, and four males aged 52, 63, 68 and 83 years, respectively. The large prevalence of immunocompromised patients in our review is an additional evidence that the rare gastric *SS* infection is a complication of systemic strongyloidiasis, either hyperinfective, or disseminated form. When investigated, duodenal co-involvement was documented in all but one case. Gastric *SS*, therefore, usually represent a hyperinfective status, a complication of duodenal infection, as shown by the data of our review (Table 1). In rare instances, gastric involvement may be secondary to oral ingestion of SS larvae, coupled with hypochlorhydria or achlorhydria.

Microscopically, adult female worms, eggs and rhabditiform larvae are seen in the epithelium of the crypts with acute or chronic inflammation in the biopsied mucosa. The degree of mucosal inflammation appears to correlate with length of infection and host response, and may be rich in eosinophils in immunocompetent individuals. Damage of the surface epithelium with hyperplastic reactive changes is noted in most of the cases. According to De Paoli and coll [18], it is the larval's entrance and exit from the parasitic tunnels that results in focal epithelial disruptions. These disruptions probably are the result of mechanical damage and possibly parasitic enzymatic activity and the action of neutrophils infiltrating the parasitic tunnels. The resulting focal erosions and ulcers are discrete mild lesions. With increased parasitic load, however, the number of lesions probably outstrips the regenerative capacity of the gut which results in coalescence of micro-ulcers and leads to villous atrophy, secondary infection and severe ulceration [18]. In contrast to adult and rhabditiform larval lesions, filariform larval lesions affect the full thickness of the gastrointestinal wall, with inflammatory reaction probably being a manifestation of allergic phenomena [18].

Our review of the literature shows that when the parasite is detected microscopically in the stomach, severe eosinophilic infiltrates are rarely observed, as opposed to duodenal mucosa. Consequently, blood eosinophilia in immunocompetent subjects is expected mostly when infection co-exists in the duodenum.

Significant male dominance (especially elderly men) has been mentioned in the literature. Data in Table 1 confirm this trend also for gastric *SS* (12 females vs 26 males; 31.6% vs 68.4%), and indicate a younger mean age in the female population (52.8 vs 57.1 years) (Table 1). As to the gastric area mostly involved by SS, our review shows that the antral-pyloric region alone (11 out of 27 cases with description) or in association with the body/fundus (14 cases) is by large the most affected area (92.6%). Gastric body involvement alone was present in just one case.

In conclusion, strongyloidiasis is a chronic and relatively asymptomatic infection of worldwide diffusion. Deadly hyperinfections may ensue when immunodepression is triggered by neoplasms or treatments for several conditions. Recent data even suggest that *SS* patients are more likely to develop several types of cancer, particularly biliary tract cancer [19]. Diligence toward the prevention of these diseases through decreased poverty and increased sanitation is mandatory. Gastric involvement is rare and almost never occurs isolated from duodenal infection. It is also most often observed in a setting of severe immunodepression. It could be facilitated by the widespread use of medications inducing inhibition of gastric acid secretion. Other causes, such as oral sex and ingestion of contaminated waters could explain primary gastric *SS* infection when there is no evidence of systemic parasitosis.

Biopsy can be used to reach a correct diagnosis of *SS* infection; however, the pathologist needs to have a wide knowledge of the types of helminthes that localise within body tissues and fluids together with the stages of development and the morphological features to avoid misdiagnosing them as arthropods or artifacts. Misdiagnoses on gastric biopsies can easily occur considering that identification of this worm in is very rare in gastric tissue.

## Data Availability

Available upon reasonable request.
